# Comprehensive risk profiling and long-term cardiovascular toxicity in HER2-positive breast cancer patients treated with trastuzumab

**DOI:** 10.3389/fonc.2025.1684289

**Published:** 2025-12-01

**Authors:** Minjing Xia, Shanshan Ding, Xueli Wang, Changdong Zhang, Song Chen, Ming Sun, Chunlin Wu, Xiong Zhang, Meiying Wang, Jia Wang, Xiaoke Shang

**Affiliations:** 1 No.2 Oncology Department, The Sixth Hospital of Wuhan, Affiliated Hospital of Jianghan University, Wuhan, Hubei, China; 2 Cancer Center, Union Hospital, Tongji Medical College, Huazhong University of Science and Technology, Wuhan, Hubei, China; 3 Laboratory of Department of Cardiovascular Surgery, Union Hospital, Tongji Medical College, Huazhong University of Science and Technology, Xi’an, Shaanxi, China; 4 Department of Cardiovascular Surgery, Union Hospital, Tongji Medical College, Huazhong University of Science and Technology, Wuhan, Hubei, China

**Keywords:** breast cancer, trastuzumab, cardiotoxicity, risk factors, left ventricular global longitudinal strain, cardiac biomarkers, cardio-oncology

## Abstract

**Objective:**

Trastuzumab-based therapy is a cornerstone for HER2-positive breast cancer but carries a risk of significant cardiotoxicity. This study aims to investigate the long-term incidence of cardiovascular adverse events (CVDs), identify a comprehensive set of risk factors, and develop a robust predictive model for trastuzumab-induced cardiotoxicity (TIC) in a well-characterized, single-center patient cohort.

**Methods:**

We retrospectively analyzed 600 HER2-positive breast cancer patients on trastuzumab-based regimens from 2018-2023. Patients were divided into CVD (n=100) and non-CVD (n=500) groups based on cardiotoxicity occurrence during a median 3.6-year follow-up. We analyzed baseline characteristics, treatment protocols, and serial monitoring data including ECG, NT-proBNP, LVEF, left ventricular global longitudinal strain (LVGLS), and cardiac biomarkers (creatine kinase (CK), CK-MB, and hs-cTnI).

**Results:**

The cumulative incidence of CVDs was 16.7%. Cardiotoxicity events included symptomatic heart failure (n=11), asymptomatic LVEF decline (n=51), significant LVGLS reduction (n=29), and significant arrhythmias (n=9). Significant baseline predictors of cardiotoxicity included age >60 years, pre-existing hyperlipidemia, and elevated NT-proBNP levels (p<0.05). Treatment with anthracycline-based chemotherapy and chest radiotherapy were also strongly associated with increased CVD risk. During follow-up, the CVD group exhibited a significantly greater decline in LVEF (baseline vs. follow-up: 64.1% vs. 48.8%) and LVGLS (-20.9% vs. -15.3%) compared to the non-CVD group (p<0.001). In multivariate logistic regression analysis, the strongest independent predictors for CVDs were a post-treatment LVEF decline >10% (OR 5.75, 95% CI 3.95-8.41), a post-treatment relative LVGLS decline >15% from baseline (OR 4.42, 95% CI 3.10-6.22), and elevated hs-cTnI (OR 4.10, 95% CI 2.91-5.74). A predictive model incorporating both baseline and on-treatment factors showed excellent discrimination (AUC = 0.88).

**Conclusion:**

Cardiotoxicity remains a major concern in long-term trastuzumab therapy. This single-center study highlights the critical importance of integrating baseline risk stratification with serial monitoring of advanced echocardiographic parameters like LVGLS and sensitive biomarkers like hs-cTnI. Our comprehensive predictive model offers a powerful tool for early identification of at-risk patients, guiding personalized surveillance and facilitating timely implementation of cardioprotective strategies to mitigate the risk of irreversible cardiac damage in this patient population.

## Introduction

Breast cancer remains the most frequently diagnosed malignancy and a primary cause of cancer-related mortality among women globally (1, 2). The amplification or overexpression of the human epidermal growth factor receptor 2 (HER2) gene, found in approximately 15-20% of breast cancers, defines an aggressive subtype with a historically poor prognosis (3, 4). The development of trastuzumab, a humanized monoclonal antibody targeting the HER2 protein, has transformed the clinical landscape for these patients. Extensive evidence confirms that trastuzumab-containing regimens significantly improve disease-free survival (DFS) and overall survival (OS) in both adjuvant and metastatic settings. For instance, in the adjuvant setting, adding trastuzumab to chemotherapy has been shown to improve 10-year OS rates from 75.2% to 84% (5, 6). In the metastatic setting, modern anti-HER2 combinations that include trastuzumab have extended the median OS to over 57 months (7, 8). Trastuzumab functions by inhibiting HER2-mediated signaling, promoting receptor degradation, and inducing antibody-dependent cell-mediated cytotoxicity (ADCC) (6, 9).

While its therapeutic benefits are well-established, a major dose-limiting toxicity of trastuzumab is cardiotoxicity, which may manifest as an asymptomatic decline in left ventricular ejection fraction (LVEF) or, less commonly, as symptomatic heart failure (10). This is classified as Type II cardiotoxicity and is generally considered reversible upon treatment cessation, unlike the irreversible myocyte damage caused by Type I agents like anthracyclines. The mechanism is linked to the blockade of the HER2 signaling pathway in cardiomyocytes, which plays an essential role in myocyte survival and stress response, particularly when challenged by other stressors like cardiotoxic chemotherapy (11).

Early detection of trastuzumab-induced cardiotoxicity (TIC) is critical to prevent permanent cardiac damage and allow for the uninterrupted continuation of life-prolonging cancer therapy. Traditionally, monitoring has depended on periodic LVEF assessment. However, LVEF is a relatively insensitive marker of early myocardial injury, as substantial damage can occur before LVEF declines (12). Consequently, more sensitive tools have been investigated. Myocardial strain imaging, particularly left ventricular global longitudinal strain (LVGLS), has emerged as a robust predictor of subsequent cardiotoxicity, often detecting subtle functional changes before an LVEF drop. Studies show that a relative reduction in GLS of 10–15% during cancer therapy is predictive of cardiotoxicity (13, 14), offering superior sensitivity over LVEF (15). Similarly, cardiac biomarkers like high-sensitivity cardiac troponin I (hs-cTnI) and N-terminal pro-B-type natriuretic peptide (NT-proBNP) have shown promise for risk stratification (16, 17). Risk stratification tools, such as the HFA-ICOS cardio-oncology risk score, have been developed to integrate various factors but require validation in diverse clinical settings (18).

While numerous studies have identified risk factors like advanced age, pre-existing cardiovascular diseases, and concurrent anthracycline use (19, 20), many were conducted in heterogeneous, multi-center populations, which can introduce variability. There is a need for studies from well-characterized, single-center cohorts with long-term follow-up to build comprehensive risk prediction models that integrate baseline factors with on-treatment monitoring parameters under a consistent clinical protocol. This study was therefore designed to leverage such a cohort to: (1) determine the long-term incidence and types of TIC, (2) comprehensively evaluate a wide array of potential risk factors, and (3) develop an integrated predictive model using modern imaging and biomarker parameters to identify patients at high risk for cardiovascular complications.

## Materials and methods

### Study design and population

**Figure 1:**
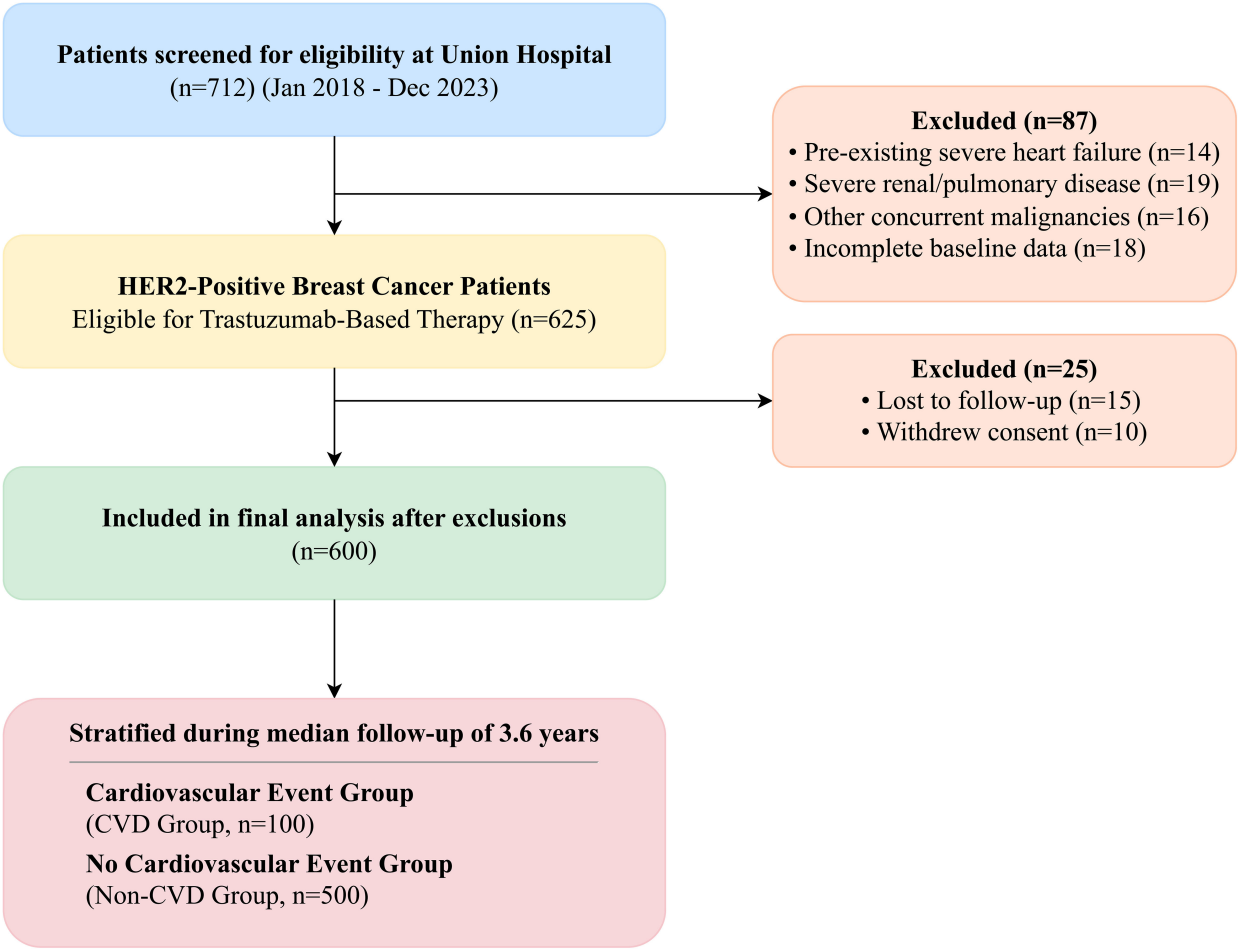
STROBE flowchart of patient selection. A total of 712 patients receiving trastuzumab for HER2-positive breast cancer were screened between January 2018 and December 2023. After applying exclusion criteria (n=87), 625 patients were included in the initial cohort. Following stratification based on follow-up events and exclusion of patients lost to follow-up or who withdrew consent (n=25), 600 patients were included in the final analysis.

We conducted a retrospective cohort study of patients with HER2-positive breast cancer who initiated trastuzumab-based therapy at Union Hospital, Tongji Medical College, between January 2018 and December 2023. This study was a consecutive enrollment of all eligible patients treated during this period, and the sample size was determined by the available patient population meeting the inclusion criteria, not by a pre-specified power calculation. A total of 712 patients were screened for eligibility from our institutional cancer registry. Patients were excluded if they had pre-existing severe heart failure (New York Heart Association [NYHA] class III-IV), severe renal dysfunction (defined as an estimated glomerular filtration rate [eGFR] < 30 mL/min/1.73m²), severe respiratory diseases such as chronic obstructive pulmonary disease or bronchial asthma, other concurrent malignancies, or incomplete baseline cardiac function data (n=87). This resulted in an initial cohort of 625 patients. A further 25 patients were excluded from the final analysis due to loss to follow-up (n=15) or withdrawal of consent (n=10), leaving a final analytical cohort of 600 patients (**Figure 1**). Patients were stratified into two groups based on the development of cardiovascular adverse events (CVD group; n=100) or no such events (non-CVD group; n=500) during the follow-up period. This study was approved by the Institutional Review Board of Union Hospital, Tongji Medical College, and the requirement for individual informed consent was waived due to its retrospective nature. The study was conducted in accordance with the Declaration of Helsinki and reported following the STROBE guidelines (21).

### Data collection and definitions

We collected comprehensive data for each patient from the institutional electronic health record system. This included: 1. Baseline Demographics and Clinical Characteristics: Age, body mass index (BMI), smoking and alcohol history, tumor stage (I-III), histological type, and comorbidities such as hypertension, diabetes, and hyperlipidemia. Baseline use of cardioprotective medications (e.g., ACE inhibitors/ARBs, beta-blockers) was also recorded. 2. Treatment Details: Chemotherapy regimen (anthracycline-based vs. non-anthracycline-based) and history of radiotherapy to the chest wall. All treatment protocols were administered according to institutional guidelines. Patients who did not receive anthracycline-based chemotherapy were typically administered a taxane and carboplatin-based protocol (e.g., docetaxel, carboplatin, and trastuzumab; TCH). The decision to omit anthracyclines was made by the treating oncologist based on clinical guidelines, primarily for patients with elevated baseline cardiovascular risk, borderline cardiac function, or advanced age, to minimize cumulative cardiotoxic effects (3, 22, 23). All patients received a standardized weight-based dosing regimen of trastuzumab for a planned duration of one year in the adjuvant setting. 3. Cardiac Monitoring: All cardiac assessments were performed at baseline (before trastuzumab initiation) and serially during follow-up, typically at 3, 6, and 12 months, and annually thereafter. The standardized protocol at our center ensured consistency in data acquisition. All echocardiographic examinations were performed by level III certified cardiologists using GE Vivid E95 systems (GE Vingmed Ultrasound, Horten, Norway). Key assessments included:

Electrocardiogram (ECG): Standard 12-lead ECGs were evaluated for ST-T changes, sinus tachycardia, and significant arrhythmias.

Echocardiography: LVEF was measured using the biplane Simpson’s method. LVGLS was assessed using 2D speckle-tracking echocardiography from apical 4-, 3-, and 2-chamber views. All echocardiograms were analyzed in a core lab by two cardiologists blinded to clinical outcomes to ensure minimal inter-observer variability (<5%).

Cardiac Biomarkers: Serum levels of CK, CK-MB, hs-cTnI, and NT-proBNP were measured using standardized assays in our central laboratory. Elevation was defined as any value exceeding the upper limit of normal (ULN) for our laboratory’s assays.

### Definition of cardiotoxicity

Our primary endpoint, trastuzumab-induced cardiotoxicity (TIC), was defined according to the 2022 ESC Guidelines on cardio-oncology (24). The composite endpoint included the occurrence of any of the following during follow-up: (1) Cancer Therapy-Related Cardiac Dysfunction (CTRCD): A new reduction in LVEF of >10 percentage points to an absolute value of <50% (symptomatic or asymptomatic); (2) Subclinical Cardiac Dysfunction: A relative percentage reduction in LVGLS of >15% from baseline; (3) Significant Arrhythmia: New-onset and sustained atrial fibrillation, ventricular tachycardia, or high-grade atrioventricular block; (4) Myocardial Injury: Elevation of cardiac hs-cTnI above the laboratory’s 99th percentile ULN. For this study, patients meeting any of these criteria were classified into the CVD group.

### Statistical analysis

All statistical analyses were performed using R software (Version 4.2.1, R Foundation for Statistical Computing, Vienna, Austria). Continuous variables are presented as mean ± standard deviation (SD) or median (interquartile range [IQR]) and compared using Student’s t-test or Mann-Whitney U test, respectively. Categorical variables are presented as frequencies and percentages and were compared using the Chi-square test or Fisher’s exact test. Kaplan-Meier analysis with the log-rank test was used to estimate and compare event-free survival. To identify independent predictors of cardiotoxicity, we performed univariate and multivariate logistic regression analyses. Variables with a p-value < 0.10 in univariate analysis or those of known clinical importance were included in the multivariate model using a backward stepwise selection method. Odds ratios (ORs) with 95% confidence intervals (CIs) were calculated. The predictive performance of key parameters and the final multivariate model was assessed using the area under the receiver operating characteristic curve (AUC). A two-sided p-value < 0.05 was considered statistically significant.

## Results

### Baseline characteristics and incidence of cardiotoxicity

A total of 600 patients were included in the final analysis. The median age of the cohort was 59 years (IQR, 50-66), and the median follow-up duration was 3.6 years (IQR, 2.2-5.1). During this period, 100 patients (16.7%) developed cardiovascular adverse events. The breakdown of events in the CVD group (n=100) was as follows: 11 patients (11.0%) developed symptomatic heart failure (NYHA Class II-IV), 51 (51.0%) had an asymptomatic decline in LVEF meeting CTRCD criteria, 29 (29.0%) had an isolated significant reduction in LVGLS (>15%), and 9 (9.0%) developed new-onset significant arrhythmias (6 atrial fibrillation, 3 ventricular tachycardia). The baseline characteristics of the CVD and non-CVD groups are detailed in **Table 1**. Patients in the CVD group were significantly older, had a higher prevalence of baseline hyperlipidemia and elevated NT-proBNP, and were more likely to have received anthracycline-based chemotherapy and chest radiotherapy (all p<0.05). There were no significant differences in baseline smoking status, alcohol use, or use of cardioprotective medications between the two groups.

**Table 1:** Baseline clinical, pathological, and treatment characteristics.

Characteristic	CVD group (n=100)	Non-CVD group (n=500)	P-value
Age (years), mean ± SD	62.1 ± 9.5	58.3 ± 10.1	<0.001
BMI (kg/m²), mean ± SD	25.1 ± 3.3	24.6 ± 3.6	0.152
Current Smoker, n (%)	14 (14.0)	61 (12.2)	0.655
Alcohol Use (>3 drinks/week), n (%)	8 (8.0)	35 (7.0)	0.761
Comorbidities, n (%)			
Hypertension	32 (32.0)	138 (27.6)	0.345
Diabetes Mellitus	18 (18.0)	71 (14.2)	0.301
Hyperlipidemia	47 (47.0)	130 (26.0)	<0.001
Use of cardioprotective agents (baseline), n (%)			
ACEi/ARB	14 (14.0)	62 (12.4)	0.689
Beta-blocker	17 (17.0)	78 (15.6)	0.724
Tumor characteristics, n (%)			
Stage I	19 (19.0)	115 (23.0)	0.518
Stage II	53 (53.0)	255 (51.0)	
Stage III	28 (28.0)	130 (26.0)	
Treatment regimens, n (%)			
Anthracycline-based Chemotherapy	66 (66.0)	228 (45.6)	<0.001
Chest Radiotherapy	54 (54.0)	190 (38.0)	0.003
Baseline cardiac markers			
LVEF (%), mean ± SD	64.1 ± 5.2	64.9 ± 4.8	0.098
LVGLS (%), mean ± SD	-20.9 ± 2.4	-21.2 ± 2.2	0.241
NT-proBNP (pg/mL), median [IQR]	160 [90-345]	95 [58-148]	<0.001

ACEi/ARB, Angiotensin-Converting Enzyme Inhibitor/Angiotensin Receptor Blocker; BMI, Body Mass Index; CVD, Cardiovascular Disease; LVEF, Left Ventricular Ejection Fraction; LVGLS, Left Ventricular Global Longitudinal Strain; NT-proBNP, N-terminal pro-B-type Natriuretic Peptide.

**Figure 2:**
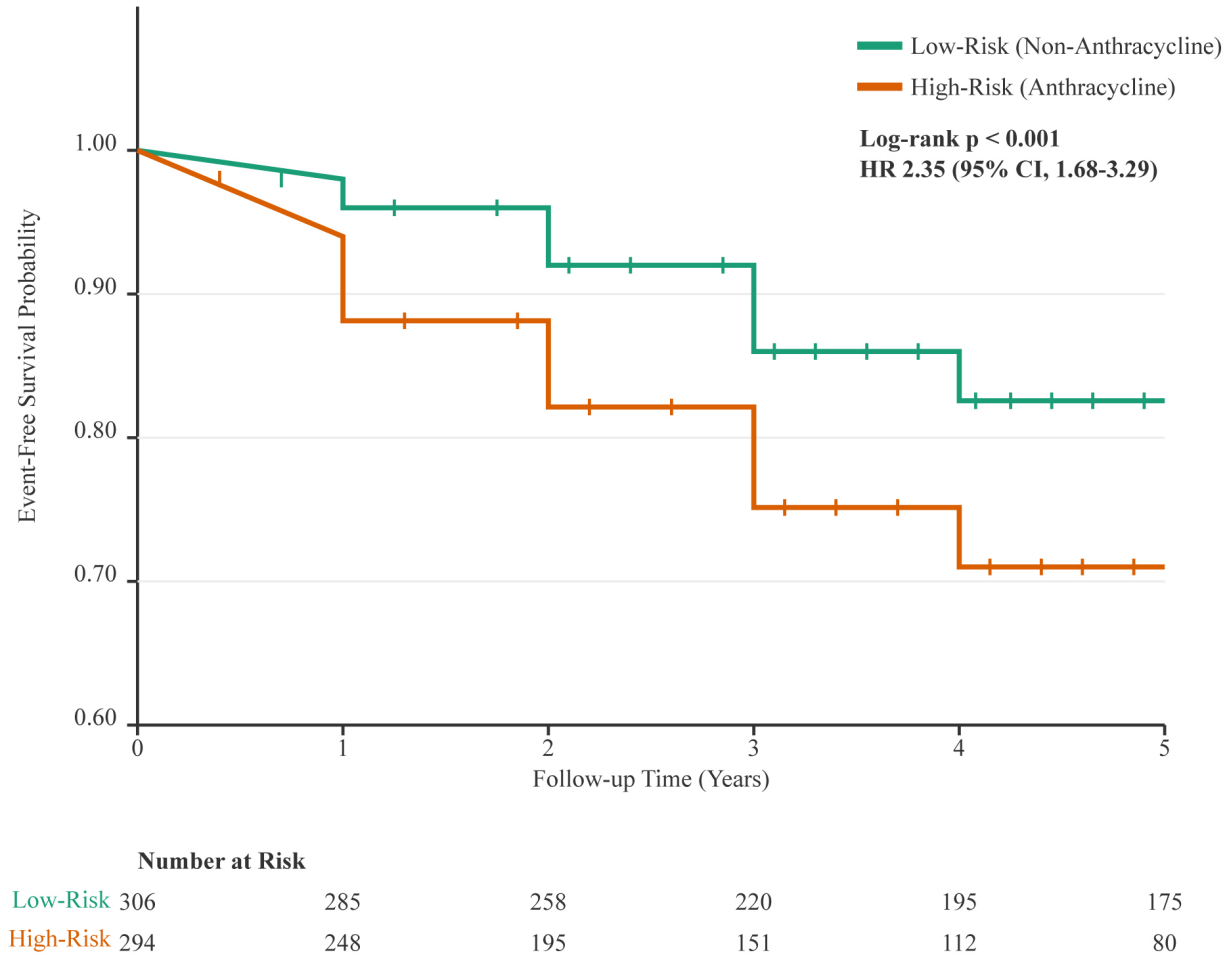
Kaplan-Meier analysis of cardiovascular event-free survival. The curve demonstrates a significantly lower probability of remaining free of cardiovascular events for patients who received anthracycline-based chemotherapy (High-Risk Group, orange line) compared to those who received non-anthracycline regimens (Low-Risk Group, green line). Vertical ticks on the curves indicate censored data points. The difference between the groups was statistically significant (log-rank p < 0.001; Hazard Ratio [HR] for anthracycline-based therapy, 2.35 [95% CI, 1.68-3.29]). The table below the plot indicates the number of patients at risk in each group at yearly intervals.

Kaplan-Meier analysis demonstrated significantly lower event-free survival in patients receiving anthracycline-based chemotherapy compared to their counterparts (log-rank p < 0.001, **Figure 2**).

### Changes in cardiac function and biomarkers during follow-up

**Figure 3:**
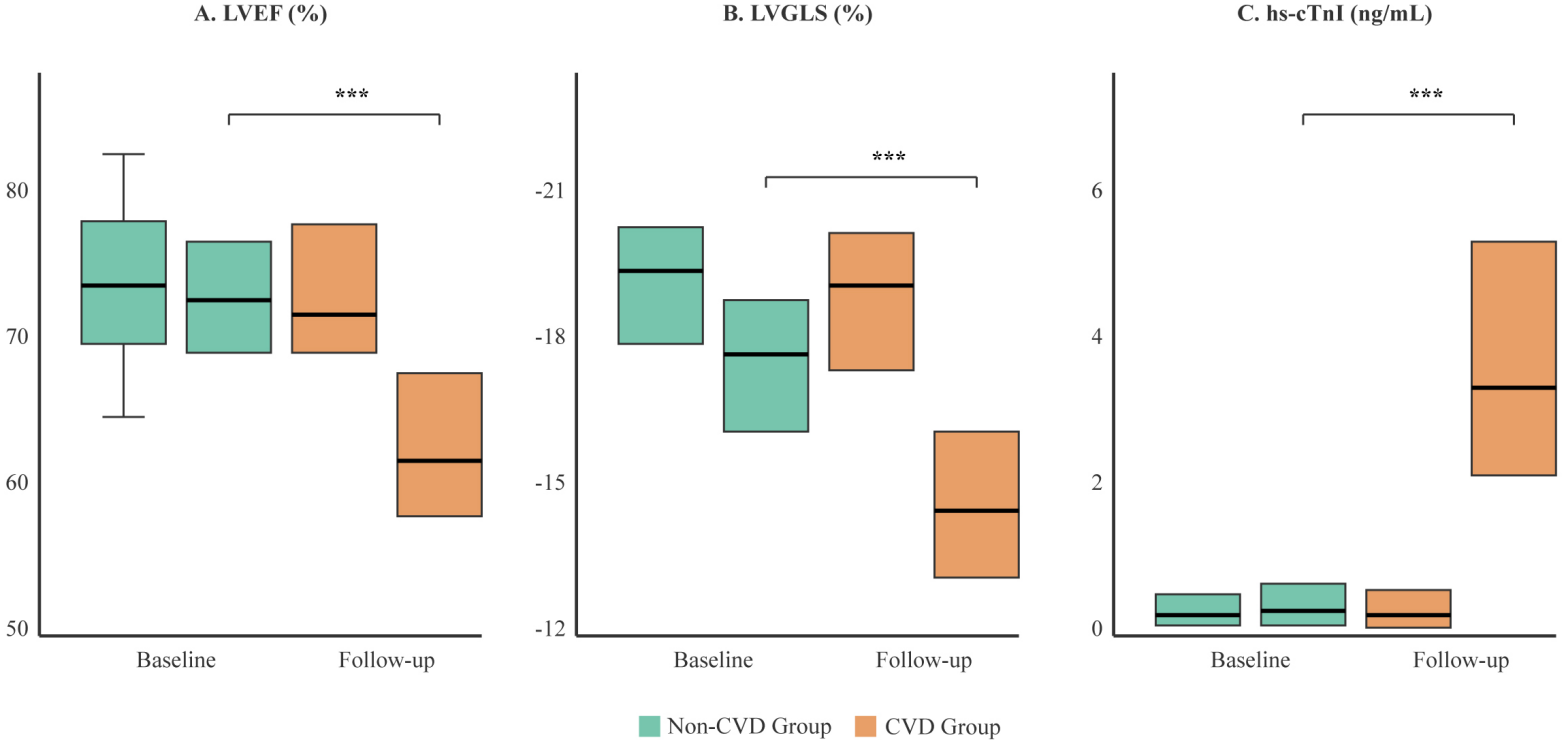
Comparison of cardiac function and biomarker changes between the CVD and non-CVD groups. Box plots illustrate the distribution of **(A)** Left Ventricular Ejection Fraction (LVEF), **(B)** Left Ventricular Global Longitudinal Strain (LVGLS), and **(C)** high-sensitivity Troponin I (hs-cTnI) at baseline and at the final follow-up. The box represents the interquartile range (IQR), the horizontal line indicates the median, and whiskers extend to 1.5 times the IQR. While baseline values were similar, the CVD group (orange) showed a significant deterioration in all parameters at follow-up compared to the non-CVD group (green). ***p < 0.001.

We observed significant differences in the evolution of cardiac parameters between the two groups (**Table 2**, **Figure 3**). At follow-up, the CVD group showed a marked decline in LVEF (mean change -15.3% vs. -2.2% in non-CVD group; p<0.001) and LVGLS (mean change +5.6% vs. +1.0% in non-CVD group; p<0.001). The absolute values at follow-up were substantially worse in the CVD group. Furthermore, patients who developed cardiotoxicity had significantly higher levels of hs-cTnI and CK-MB post-treatment. ECG abnormalities, particularly new ST-T changes and new-onset arrhythmias, were also far more prevalent in the CVD group after therapy initiation, as detailed in **Table 2**.

**Table 2:** Comparison of cardiac monitoring parameters at baseline and follow-up.

Parameter	Time point	CVD group (n=100)	Non-CVD group (n=500)	P-value
LVEF (%), mean ± SD	Baseline	64.1 ± 5.2	64.9 ± 4.8	0.098
	Follow-up	48.8 ± 8.5	62.7 ± 5.4	<0.001
LVGLS (%), mean ± SD	Baseline	-20.9 ± 2.4	-21.2 ± 2.2	0.241
	Follow-up	-15.3 ± 3.2	-20.2 ± 2.9	<0.001
hs-cTnI (ng/L), median [IQR]	Baseline	4.1 [2.5-6.0]	3.8 [2.2-5.5]	0.071
	Follow-up	15.2 [9.8-25.1]	4.5 [2.8-6.4]	<0.001
CK-MB (U/L), median [IQR]	Baseline	8.2 [7.0-9.5]	8.1 [6.8-9.3]	0.612
	Follow-up	16.5 [14.0-18.9]	8.9 [7.5-10.1]	<0.001
ST-T Changes, n (%)	Baseline	6 (6.0)	22 (4.4)	0.520
	Follow-up	49 (49.0)	41 (8.2)	<0.001
New-onset Arrhythmia, n (%)	Follow-up	9 (9.0)	5 (1.0)	<0.001

### Predictors of cardiovascular toxicity

**Figure 4:**
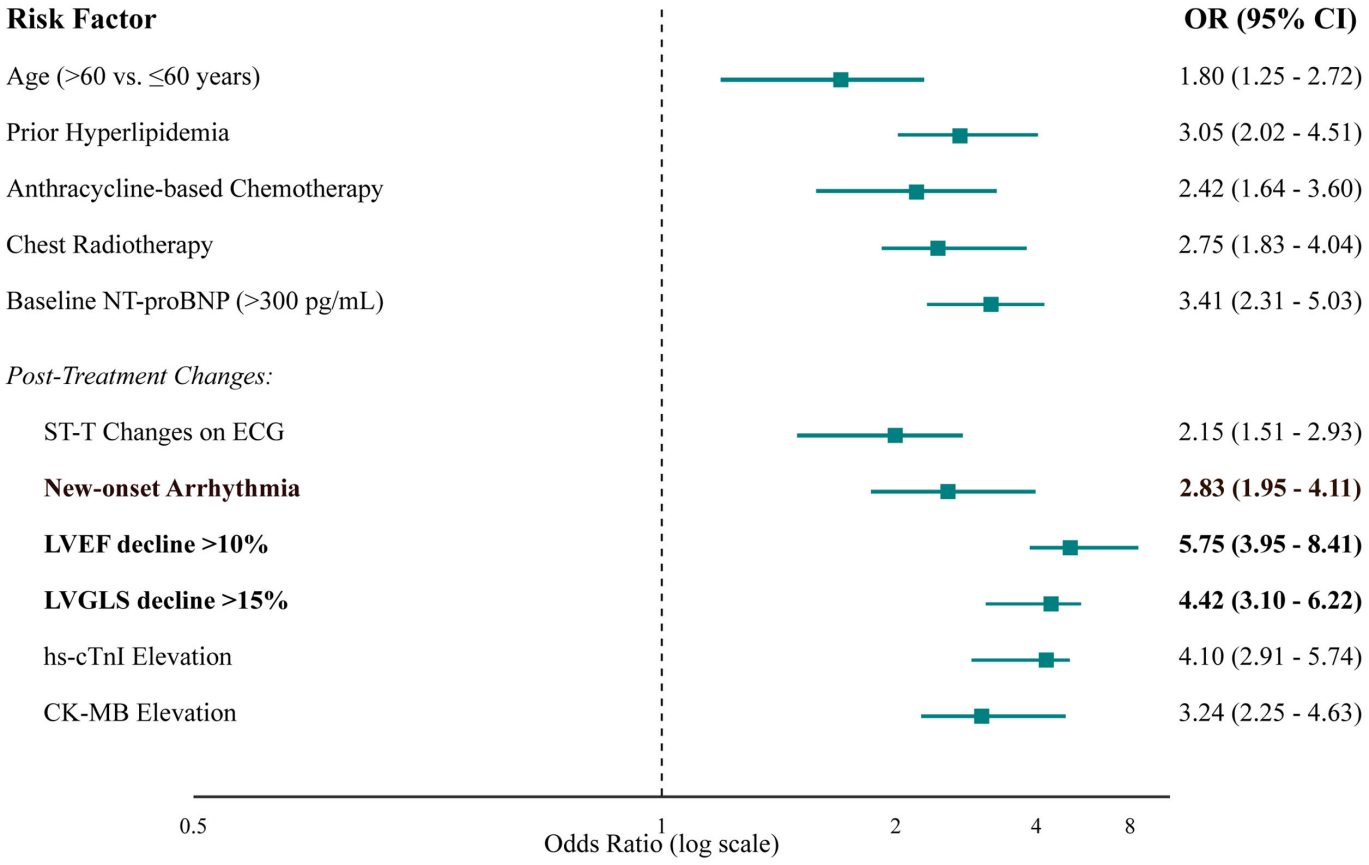
Forest plot of independent predictors of cardiovascular events from multivariate logistic regression analysis. The plot displays the adjusted odds ratios (squares) and their corresponding 95% confidence intervals (horizontal lines). The size of the square is proportional to the precision of the estimate. The vertical dashed line at an OR of 1 represents no effect. Factors with CIs that do not cross this line are statistically significant predictors of cardiotoxicity.

After adjusting for confounders in a multivariate logistic regression model, several factors remained strong, independent predictors (**Table 3**, **Figure 4**). Key baseline predictors were prior hyperlipidemia (OR 3.05, 95% CI 2.02-4.51) and use of an anthracycline-based regimen (OR 2.42, 95% CI 1.64-3.60). However, the most powerful predictors were on-treatment changes in cardiac monitoring parameters. A decline in LVEF of >10% (OR 5.75, 95% CI 3.95-8.41) and a relative decline in LVGLS of >15% (OR 4.42, 95% CI 3.10-6.22) were the strongest predictors of subsequent clinical events. Elevated cardiac biomarkers, particularly hs-cTnI, were also highly predictive (OR 4.10, 95% CI 2.91-5.74).

**Table 3:** Univariate and multivariate logistic regression analysis for predictors of cardiovascular events.

Risk factor	Univariate OR (95% CI)	P-value	Multivariate adjusted OR (95% CI)	P-value
Baseline factors				
Age > 60 years	2.05 (1.38 - 3.04)	<0.001	1.80 (1.25 - 2.72)	0.002
Prior Hyperlipidemia	3.41 (2.29 - 5.09)	<0.001	3.05 (2.02 - 4.51)	<0.001
Anthracycline-based Chemo	2.78 (1.89 - 4.08)	<0.001	2.42 (1.64 - 3.60)	<0.001
Chest Radiotherapy	2.92 (2.01 - 4.25)	<0.001	2.75 (1.83 - 4.04)	<0.001
Baseline NT-proBNP > 300pg/mL	3.66 (2.53 - 5.29)	<0.001	3.41 (2.31 - 5.03)	<0.001
On-treatment dynamic factors				
ST-T Changes on ECG	2.41 (1.70 - 3.42)	<0.001	2.15 (1.51 - 2.93)	<0.001
New-onset Arrhythmia	9.82 (3.51 - 27.5)	<0.001	2.83 (1.95 - 4.11)	<0.001
LVEF decline > 10%	6.23 (4.31 - 9.01)	<0.001	5.75 (3.95 - 8.41)	<0.001
LVGLS relative decline > 15%	5.05 (3.58 - 7.12)	<0.001	4.42 (3.10 - 6.22)	<0.001
hs-cTnI Elevation > ULN	4.61 (3.30 - 6.44)	<0.001	4.10 (2.91 - 5.74)	<0.001
CK-MB Elevation > ULN	3.68 (2.61 - 5.18)	<0.001	3.24 (2.25 - 4.63)	<0.001

Adjusted model includes all listed variables. Hosmer-Lemeshow test P = 0.41, indicating good model fit. ULN, Upper Limit of Normal.

### Predictive value of cardiac monitoring models and clinical outcomes

**Figure 5:**
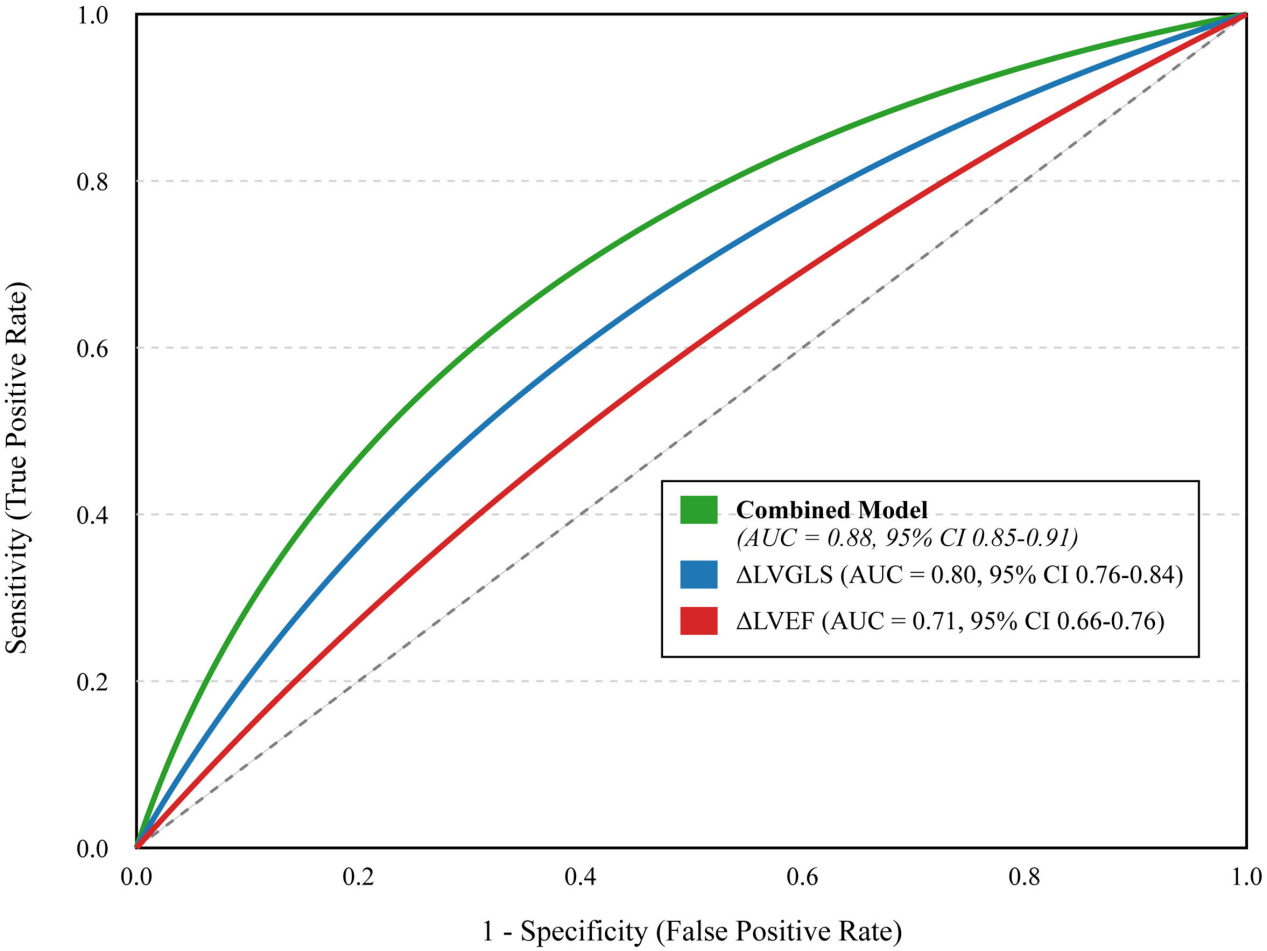
Receiver Operating Characteristic (ROC) curves for different predictive models. The curves illustrate the diagnostic ability of a model based on post-treatment change in LVEF alone (ΔLVEF, red line), a model based on post-treatment change in LVGLS alone (ΔLVGLS, blue line), and a comprehensive combined model including baseline risk factors and on-treatment changes in both imaging and biomarkers (Combined Model, green line). The area under the curve (AUC) with 95% confidence intervals is provided for each model. The diagonal dashed line represents a test with no discriminatory power (AUC = 0.5). The “Combined Model” incorporates significant baseline risk factors (age >60, hyperlipidemia, anthracycline/radiotherapy use, NT-proBNP >300 pg/mL) and on-treatment changes (LVEF decline >10%, LVGLS decline >15%, new-onset arrhythmia, hs-cTnI elevation).

We assessed the ability of different models to predict CVD events using ROC analysis (**Figure 5**). A model using only the change in LVEF had moderate predictive power (AUC = 0.71). Incorporating the change in LVGLS significantly improved prediction (AUC = 0.80). A comprehensive model that combined baseline clinical risk factors (age, hyperlipidemia, anthracycline/radiotherapy use) with on-treatment changes in LVGLS, LVEF, and hs-cTnI demonstrated excellent discriminatory power, with an AUC of 0.88 (95% CI 0.85-0.91). Furthermore, patients who developed cardiotoxicity experienced significantly worse long-term clinical outcomes, including higher rates of heart failure-related re-hospitalization (21.0% vs. 1.2%, p < 0.001) and all-cause mortality (15.0% vs. 3.4%, p < 0.001) during the follow-up period (**Table 4**).

**Table 4:** Clinical outcomes at final follow-up.

Outcome	CVD group (n=100)	Non-CVD group (n=500)	P-value
Heart Failure-Related Re-hospitalization, n (%)	21 (21.0)	6 (1.2)	<0.001
All-Cause Mortality, n (%)	15 (15.0)	17 (3.4)	<0.001

## Discussion

In this single-center study with longitudinal follow-up, we provide a detailed evaluation of the incidence and predictors of trastuzumab-induced cardiotoxicity. Our findings confirm that TIC is a significant clinical issue, affecting 16.7% of patients in our cohort, a rate consistent with previous large-scale registries but derived from a population with highly standardized monitoring protocols (25, 26). The key contribution of this work is the development of an integrated risk model in a homogenous cohort, which confirms that a multi-parameter approach combining baseline risk factors with dynamic on-treatment changes offers superior predictive accuracy for clinical events.

Our results reinforce the well-established role of concomitant cardiotoxic therapies, with both anthracycline use and chest radiotherapy emerging as major risk factors (20, 27). This highlights the “multiple-hit” hypothesis, where prior or concurrent myocardial stress renders the heart more susceptible to HER2 blockade (27). The significant association with baseline hyperlipidemia and elevated NT-proBNP suggests that subclinical cardiovascular vulnerability predisposes patients to TIC. This underscores the importance of a thorough baseline cardiovascular risk assessment as recommended by current cardio-oncology guidelines (24, 28).

The standout finding of our study is the superior predictive value of dynamic monitoring markers. It is important to distinguish between baseline predictors and on-treatment markers. Baseline factors such as age, comorbidities like hyperlipidemia, and planned treatments like anthracycline use serve as true predictors of future risk before therapy initiation. In contrast, on-treatment changes, such as a >15% relative drop in LVGLS from baseline, function as powerful real-time markers indicating subclinical myocardial injury is actively occurring. This decline in LVGLS was a powerful predictor of cardiotoxicity, consistent with a growing body of evidence (29–31). LVGLS detects subtle myocardial dysfunction before changes in LVEF occur, offering a crucial window for intervention. While LVGLS measurements can be subject to inter-operator and inter-vendor variability, these limitations were minimized in our study through standardized equipment and assessment by experienced, blinded core lab cardiologists (32). Other advanced echocardiographic parameters, such as left ventricular (LV) diastolic function and volumes, also provide valuable prognostic information but were not systematically included in our primary analysis, representing an area for future investigation. Similarly, the elevation of hs-cTnI post-treatment was a strong predictor, signifying myocyte injury, a finding that aligns with previous landmark studies (26, 33). Our comprehensive model, achieving an AUC of 0.88, illustrates the power of a multi-parameter approach for personalized risk stratification.

A crucial clinical implication from this study is the potential to guide the timely initiation of cardioprotective therapies. Although baseline use of agents like ACE inhibitors or beta-blockers was not associated with a lower incidence of TIC in our cohort, likely due to confounding by indication—wherein patients with higher baseline cardiovascular risk are more likely to be prescribed these medications, thus masking their potential benefits in statistical analysis—our predictive model allows for the early identification of high-risk patients. For these individuals, preemptive initiation of such therapies upon detection of early subclinical markers (e.g., a drop in LVGLS or rise in hs-cTnI) could mitigate or prevent irreversible cardiac damage, thereby allowing the safe continuation of essential cancer treatment (34, 35). This proactive approach represents a key strategy in modern cardio-oncology care.

The major strengths of our study include the detailed, longitudinal data from a well-characterized cohort managed at a single institution. This design ensures high uniformity in diagnostic criteria and data collection. However, several limitations must be acknowledged. First, the retrospective nature introduces potential for selection bias. Second, findings from a single center may not be generalizable to other populations. Additionally, while hypertension is a known cardiovascular risk factor, patients with controlled hypertension were not excluded to ensure the study population reflects real-world clinical practice, as hypertension is a common comorbidity. Its effect was statistically adjusted for in the multivariate analysis. Third, while we adjusted for a wide range of confounders, residual confounding, particularly in differentiating the cardiotoxic effects of trastuzumab from those of concurrent chemotherapy and radiotherapy, cannot be entirely excluded. A comparative cohort of HER2-negative patients receiving similar chemotherapy would be required to fully isolate trastuzumab’s specific impact. This challenge, however, reflects the clinical reality where patients are exposed to multiple cardiotoxic insults. Therefore, our comprehensive model, which integrates various risk factors, is pragmatically designed to predict the net risk in such real-world scenarios rather than isolating the effect of a single agent. Furthermore, as patients received standardized dosing of trastuzumab, this study was not designed to assess a dose- or duration-dependent relationship for TIC, which could be an area for future investigation. Finally, our cohort size of 600 patients, while robust for a single-center study, means the number of patients with events (n=100) is modest, and our predictive model requires external validation in larger, prospective multi-center studies.

## Conclusion

In conclusion, trastuzumab-induced cardiotoxicity is a frequent complication in the long-term management of HER2-positive breast cancer. Our single-center experience provides strong evidence that a monitoring strategy incorporating baseline risk factors with serial assessment of LVGLS and hs-cTnI is superior for early identification of at-risk patients. This risk-stratified approach can facilitate timely cardioprotective interventions to improve long-term cardiovascular outcomes without compromising oncologic efficacy.

## Data Availability

The original contributions presented in the study are included in the article/supplementary material. Further inquiries can be directed to the corresponding authors.

## References

[1] Sung H Ferlay J Siegel RL . Global cancer statistics 2020: GLOBOCAN estimates of incidence and mortality worldwide for 36 cancers in 185 countries. CA Cancer J Clin. (2021) 71:209–49. doi: 10.3322/caac.21660, PMID: 33538338

[2] Wilkinson L Gathani T . Understanding breast cancer as a global health concern. Br J Radiol. (2022) 95:20211033. doi: 10.1259/bjr.20211033, PMID: 34905391 PMC8822551

[3] Loibl S Gianni L . HER2-positive breast cancer. Lancet. (2017) 389:2415–29. doi: 10.1016/s0140-6736(16)32417-5, PMID: 27939064

[4] Kunte S Abraham J Montero AJ . Novel HER2-targeted therapies for HER2-positive metastatic breast cancer. Cancer. (2020) 126:4278–88. doi: 10.1002/cncr.33102, PMID: 32721042

[5] Cicin İ. Oukkal M . An open-label, multinational, multicenter, phase IIIb study with subcutaneous administration of trastuzumab in patients with HER2-positive early breast cancer to evaluate patient satisfaction. Eur J Breast Health. (2022) 18:63–73. doi: 10.4274/ejbh.galenos.2021.2021-9-9, PMID: 35059593 PMC8734518

[6] Hensing W Santa-Maria CA Peterson LL Sheng JY . Landmark trials in the medical oncology management of early stage breast cancer. Semin Oncol. (2020) 47:278–92. doi: 10.1053/j.seminoncol.2020.08.001, PMID: 32933761 PMC7655597

[7] Slamon DJ Leyland-Jones B Shak S Fuchs H Paton V Bajamonde A . Use of chemotherapy plus a monoclonal antibody against HER2 for metastatic breast cancer that overexpresses HER2. N Engl J Med. (2001) 344:783–92. doi: 10.1056/nejm200103153441101, PMID: 11248153

[8] Schettini F Prat A . Dissecting the biological heterogeneity of HER2-positive breast cancer. Breast. (2021) 59:339–50. doi: 10.1016/j.breast.2021.07.019, PMID: 34392185 PMC8374722

[9] Valabrega G Montemurro F Aglietta M . Trastuzumab: mechanism of action, resistance and future perspectives in HER2-overexpressing breast cancer. Ann Oncol. (2007) 18:977–84. doi: 10.1093/annonc/mdl475, PMID: 17229773

[10] Zamorano JL Lancellotti P Rodriguez Muñoz D Aboyans V Asteggiano R Galderisi M . 2016 ESC Position Paper on cancer treatments and cardiovascular toxicity developed under the auspices of the ESC Committee for Practice Guidelines: The Task Force for cancer treatments and cardiovascular toxicity of the European Society of Cardiology (ESC). Eur Heart J. (2016) 37:2768–801. doi: 10.1093/eurheartj/ehw211, PMID: 27567406

[11] Nardone V Falivene S Giugliano FM Gaetano M Giordano P Muto M . The role of radiation therapy and systemic therapies in elderly with breast cancer. Transl Cancer Res. (2020) 9:S97–s109. doi: 10.21037/tcr.2019.07.04, PMID: 35117951 PMC8798854

[12] Plana JC Galderisi M Barac A Ewer MS Ky B Scherrer-Crosbie M . Expert consensus for multimodality imaging evaluation of adult patients during and after cancer therapy: a report from the American Society of Echocardiography and the European Association of Cardiovascular Imaging. J Am Soc Echocardiogr. (2014) 27:911–39. doi: 10.1016/j.echo.2014.07.012, PMID: 25172399

[13] Brady B King G Murphy RT . Myocardial strain: a clinical review. Ir J Med Sci. (2023) 192:1649–56. doi: 10.1007/s11845-022-03210-8, PMID: 36380189 PMC9666989

[14] van der Linde D van Hagen I Veen K Zuetenhorst H van Dalen B . Global longitudinal strain: an early marker for cardiotoxicity in patients treated for breast cancer. Neth Heart J. (2023) 31:103–8. doi: 10.1007/s12471-022-01734-3, PMID: 36434383 PMC9950304

[15] Venneri L Khattar RS . Cancer and myocardial dysfunction: is there a link? Expert Rev Cardiovasc Ther. (2016) 14:1207–9. doi: 10.1080/14779072.2016.1226129, PMID: 27538574

[16] Sulaiman L Hesham D Abdel Hamid M Youssef G . The combined role of NT-proBNP and LV-GLS in the detection of early subtle chemotherapy-induced cardiotoxicity in breast cancer female patients. Egypt Heart J. (2021) 73:20. doi: 10.1186/s43044-021-00142-z, PMID: 33649999 PMC7921250

[17] Mihalcea D Memis H Mihaila S Vinereanu D . Cardiovascular toxicity induced by vascular endothelial growth factor inhibitors. Life (Basel). (2023) 13. doi: 10.3390/life13020366, PMID: 36836722 PMC9965690

[18] Cronin M Crowley A Davey MG . Heart failure association-international cardio-oncology society risk score validation in HER2-positive breast cancer. J Clin Med. (2023) 12. doi: 10.3390/jcm12041278, PMID: 36835818 PMC9963986

[19] Chang WT Chen PW Lin HW Lin SH Li YH . Risks of trastuzumab-related cardiotoxicity in breast cancer patients in Taiwan. ESC Heart Fail. (2021) 8:5149–58. doi: 10.1002/ehf2.13591, PMID: 34480791 PMC8712795

[20] Kamphuis JAM Linschoten M Cramer MJ Doevendans PA Asselbergs FW Teske AJ . Early- and late anthracycline-induced cardiac dysfunction: echocardiographic characterization and response to heart failure therapy. Cardiooncology. (2020) 6:23. doi: 10.1186/s40959-020-00079-3, PMID: 33072403 PMC7557080

[21] von Elm E Altman DG Egger M . The Strengthening the Reporting of Observational Studies in Epidemiology (STROBE) statement: guidelines for reporting observational studies. J Clin Epidemiol. (2007) 60:344–9. doi: 10.1136/bmj.39335.541782.AD, PMID: 18313558

[22] Untch M . Targeted therapy for early and locally advanced breast cancer. Breast Care (Basel). (2010) 5:144–52. doi: 10.1159/000315047, PMID: 20847827 PMC2931052

[23] Costa RB Kurra G Greenberg L Geyer CE . Efficacy and cardiac safety of adjuvant trastuzumab-based chemotherapy regimens for HER2-positive early breast cancer. Ann Oncol. (2010) 21:2153–60. doi: 10.1093/annonc/mdq096, PMID: 20351072

[24] 2022 ESC Guidelines on cardio-oncology developed in collaboration with the European Hematology Association (EHA), the European Society for Therapeutic Radiology and Oncology (ESTRO) and the International Cardio-Oncology Society (IC-OS). G Ital Cardiol (Rome). (2023) 24:e1–e130. doi: 10.1714/3967.39463, PMID: 36853174

[25] Castillo C Camejo N Etcheverria C Ferradaz J Ferreira A Fontan A . Trastuzumab-induced cardiotoxicity in early breast cancer over a 10-year period in Uruguay. Med (Baltimore). (2022) 101:e29927. doi: 10.1097/md.0000000000029927, PMID: 35905268 PMC9333491

[26] Ky B Putt M Sawaya H French B Januzzi JL Jr. Sebag IA . Early increases in multiple biomarkers predict subsequent cardiotoxicity in patients with breast cancer treated with doxorubicin, taxanes, and trastuzumab. J Am Coll Cardiol. (2014) 63:809–16. doi: 10.1016/j.jacc.2013.10.061, PMID: 24291281 PMC4286181

[27] Zhu Q Kirova YM Cao L Arsene-Henry A Chen J . Cardiotoxicity associated with radiotherapy in breast cancer: A question-based review with current literatures. Cancer Treat Rev. (2018) 68:9–15. doi: 10.1016/j.ctrv.2018.03.008, PMID: 29777800

[28] Herrmann J Lenihan D . Defining cardiovascular toxicities of cancer therapies: an International Cardio-Oncology Society (IC-OS) consensus statement. Eur Heart J. (2022) 43:280–99. doi: 10.1093/eurheartj/ehab674, PMID: 34904661 PMC8803367

[29] Thavendiranathan P Poulin F Lim KD Plana JC Woo A Marwick TH . Use of myocardial strain imaging by echocardiography for the early detection of cardiotoxicity in patients during and after cancer chemotherapy: a systematic review. J Am Coll Cardiol. (2014) 63:2751–68. doi: 10.1016/j.jacc.2014.01.073, PMID: 24703918

[30] Glen C Tan YY Waterston A Evans TRJ Jones RJ Petrie MC . Mechanistic and clinical overview cardiovascular toxicity of BRAF and MEK inhibitors: JACC: cardioOncology state-of-the-art review. JACC CardioOncol. (2022) 4:1–18. doi: 10.1016/j.jaccao.2022.01.096, PMID: 35492830 PMC9040125

[31] Sławiński G Hawryszko M Liżewska-Springer A I. Nabiałek-Trojanowska and E . Lewicka: global longitudinal strain in cardio-oncology: A review. Cancers (Basel). (2023) 15. doi: 10.3390/cancers15030986, PMID: 36765941 PMC9913863

[32] Spitzer E Camacho B . Echocardiography core laboratory validation of a novel vendor-independent web-based software for the assessment of left ventricular global longitudinal strain. J Cardiovasc Imaging. (2023) 31:135–41. doi: 10.4250/jcvi.2022.0130, PMID: 37488918 PMC10374390

[33] Sandhu H Maddock H . Molecular basis of cancer-therapy-induced cardiotoxicity: introducing microRNA biomarkers for early assessment of subclinical myocardial injury. Clin Sci (Lond). (2014) 126:377–400. doi: 10.1042/cs20120620, PMID: 24274966

[34] Haj-Yehia E Michel L Mincu RI Rassaf T Totzeck M . Prevention of cancer-therapy related cardiac dysfunction. Curr Heart Fail Rep. (2025) 22:9. doi: 10.1007/s11897-025-00697-x, PMID: 39969700 PMC11839799

[35] Patel R Zarifa A Bajwa T Lee K Robin J Saha P . Use of cardiac biomarkers to improve initiation of cardioprotective therapy in patients with breast cancer receiving cardiotoxic chemotherapy in a multidisciplinary cancer center. JCO Oncol Pract. (2024) 20:344–4. doi: 10.1200/OP.2024.20.10_suppl.344

